# Disturbed alternative splicing of FIR (PUF60) directed cyclin E overexpression in esophageal cancers

**DOI:** 10.18632/oncotarget.25149

**Published:** 2018-05-01

**Authors:** Yukiko Ogura, Tyuji Hoshino, Nobuko Tanaka, Guzhanuer Ailiken, Sohei Kobayashi, Kouichi Kitamura, Bahityar Rahmutulla, Masayuki Kano, Kentarou Murakami, Yasunori Akutsu, Fumio Nomura, Sakae Itoga, Hisahiro Matsubara, Kazuyuki Matsushita

**Affiliations:** ^1^ Department of Frontier Surgery, Graduate School of Medicine, Chiba University, Chiba, Japan; ^2^ Department of Physical Chemistry, Graduate School of Pharmaceutical Sciences, Chiba University, Chiba, Japan; ^3^ Department of Laboratory Medicine & Division of Clinical Genetics and Proteomics, Chiba University Hospital, Chiba, Japan; ^4^ Department of Molecular Diagnosis, Graduate School of Medicine, Chiba University, Chiba, Japan; ^5^ Department of Molecular Oncology, Graduate School of Medicine, Chiba University, Chiba, Japan

**Keywords:** esophageal squamous cell carcinoma (ESCC), alternative splicing (AS), FBW7, FIR (PUF60), cyclin E

## Abstract

Overexpression of alternative splicing of far upstream element binding protein 1 (FUBP1) interacting repressor (FIR; poly(U) binding splicing factor 60 [PUF60]) and cyclin E were detected in esophageal squamous cell carcinomas (ESCC). Accordingly, the expression of FBW7 was examined by which cyclin E is degraded as a substrate via the proteasome system. Expectedly, FBW7 expression was decreased significantly in ESCC. Conversely, *c-myc* gene transcriptional repressor FIR (alias PUF60; U2AF-related protein) and its alternative splicing variant form (FIRΔexon2) were overexpressed in ESCC. Further, anticancer drugs (cis-diaminedichloroplatinum/cisplatin [CDDP] or 5-fluorouracil [5-FU]) and knockdown of FIR by small interfering RNA (siRNA) increased cyclin E while knockdown of FIRΔexon2 by siRNA decreased cyclin E expression in ESCC cell lines (TE1, TE2, and T.Tn) or cervical SCC cells (HeLa cells). Especially, knockdown of SAP155 (SF3b1), a splicing factor required for proper alternative splicing of FIR pre-mRNA, decreased cyclin E. Therefore, disturbed alternative splicing of FIR generated FIR/FIRΔexon2 with cyclin E overexpression in esophageal cancers, indicating that SAP155 siRNA potentially rescued FBW7 function by reducing expression of FIR and/or FIRΔexon2. Remarkably, Three-dimensional structure analysis revealed the hypothetical inhibitory mechanism of FBW7 function by FIR/FIRΔexon2, a novel mechanism of cyclin E overexpression by FIR/FIRΔexon2-FBW7 interaction was discussed. Clinically, elevated FIR expression potentially is an indicator of the number of lymph metastases and anti-FIR/FIRΔexon2 antibodies in sera as cancer diagnosis, indicating chemical inhibitors of FIR/FIRΔexon2-FBW7 interaction could be potential candidate drugs for cancer therapy. In conclusion, elevated cyclin E expression was, in part, induced owing to potential FIR/FIRΔexon2–FBW7 interaction in ESCC.

## INTRODUCTION

Unlike Western populations, esophageal cancer among Japanese populations consists predominately of squamous cell carcinoma (SCC). Esophageal SCC (ESCC) has frequent TP53 mutations independent of c-Myc activation. Therefore, revealing the dysregulated DNA damage response mechanism is required for understanding of ESCC carcinogenesis [1, 2]. The difficulty of ESCC treatment is due to frequent metastasis into organs and lymph nodes, as well as strong side effects or resistance to anticancer drugs/radiation [3]. Recently, DNA damage has been reported to induce alternative splicing (AS), [4] but the significance of AS in carcinogenesis remains largely unclear [5, 6]. Far upstream element binding protein 1 (FUBP1), a transcriptional activator of the *c-myc* gene, is activated in many cancers [7, 8]. FUBP1-interacting repressor (FIR) is a *c-myc* gene transcriptional repressor [9] and is a splicing variant of poly(U) binding splicing factor 60 (PUF60) [5]. FIR/PUF60 is a multifunctional protein through AS, engaging in *c-myc* gene transcriptional repression, [10] RNA splicing, [5] and DNA damage repair [4]. In cancers, exon2 of FIR, containing a transcriptional repressor site, is missing due to AS (FIRΔexon2) [10-14]. Splicing factors SAP155 (SF3b1) are required for proper AS of FIR pre-mRNA [4, 11]. The inhibition of FIR-SAP155 interaction leads to an abnormally spliced form of FIRs, which affects the regulation of *c-myc*, resulting in cell proliferation and death [15]. On the other hand, F-box and WD repeat domain-containing 7 (FBW7) frequently is mutated in hematopoietic tumors [16]. FBW7 is a member of the Skp1-Cull-F-box (SCF) type ubiquitin ligase complex and is involved in degradation of various growth-related proteins, Notch1, c-Myc, c-Jun, and cyclin E via the proteasome system [17-19]. Differential regulation of FBW7 isoform can be induced by various stimuli, [20] and altered expression of FBW7 has been reported in many blood diseases [16, 21]. The interaction between FBW7 and cis-diaminedichloroplatinum: cisplatin (CDDP) increases the cytotoxicity in non-small cell lung cancer [22]. In this study, the expression of FBW7, FIR, and cyclin E in human excised ESCC was examined in association with clinical significance, lymph node metastasis, and prognosis or treatment response. A novel mechanism of cyclin E overexpression by possible FIRs-FBW7 interaction is also discussed.

## RESULTS

### Cyclin E, TP53, and FIR were upregulated whereas FBW7 was downregulated in ESCC tissues

**Figure 1:**
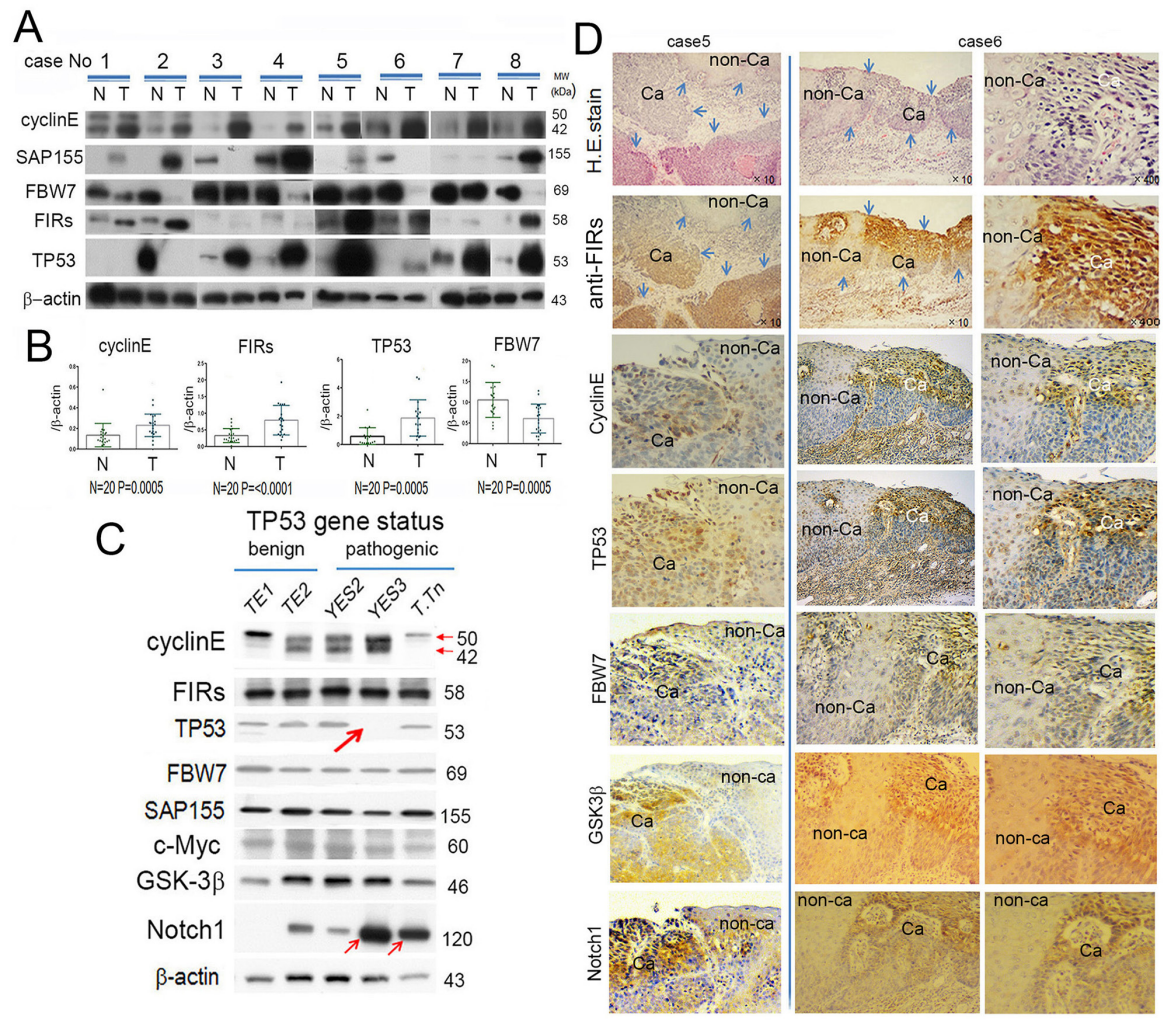
FBW7 was significantly decreased whereas FIRs, cyclin E and TP53 were significantly increased in esophageal cancer tissues indicated by Western blot analysis **(A)** Western blot analysis of cyclin E, SAP155, FBW7, FIRs, and TP53 expression in esophageal cancer tissue (T) and non-cancer tissues (N) without chemoradiation therapy before operation. β-actin was used as internal control. Cyclin E, FIRs, and TP53 were significantly increased in cancer tissues than in non-cancer tissues. On the other hand, FBW7 expression was significantly decreased in cancer tissue. Note, in case 2, TP53 was paradoxically highly expressed in (N) than that of (T). **(B)** Histogram of protein expression between cancer and non-cancer tissues. The ratio of qRT-PCR results were cyclin E (T/N)=2.0, FIRs (T/N)=2.6, TP53 (T/N)=3.3 and FBW7 (T/N)=0.54. Statistical analysis was performed by *t*-test. *P* values < 0.05 were considered significant. **(C)** Correlation of T/N ration between TP53 and FBW7 was indicated. There was weak negative correlation between TP53 (T/N) and FBW7 (T/N). **(D)** Cyclin E, FIRs, TP53, FBW7, SAP155, c-Myc, GSK-3β, and Notch1 expression were indicated depending on the TP53 mutational status. β-actin was used as an internal endogenous control. TP53 variation status was benign in HeLa, TE1 and TE2 whereas pathogenic in YES, YES3 and T.Tn. The TP53 was truncated in YES3 cells (thick arrow). Cyclin E was less expressed whereas Notch 1 was increased in YES3 and T.Tn, than those of benign cells (thin arrows). FIR, cyclin E, TP53, FBW7, GSK-3β, Notch1 expression and H&E staining was performed by immunohistochemical staining in esophageal cancer (Ca) and corresponding non-cancer tissues (non-Ca).

Cyclin E, FIRs (sum of authentic FIR and FIRΔexon2, an alternative splicing form of FIR) and TP53 expression were significantly increased whereas FBW7 was significantly decreased in ESCC tissues than in those of corresponding non-cancer tissues (Figure 1A, 1B). Weak negative correlation was found in T/N ratio between TP53 and FBW7 (Figure 1C). ***TP53*** gene variation of TE1 and TE2 was benign (wild) whereas that of YES2 and T.Tn was pathogenic, however, no significant difference of TP53 expression among these cells (Figure 1D, Supplementary Figure 2). *TP53* gene mutation in YES3 generated truncated TP53 or scarce expression (Figure 1D, large arrow, Supplementary Figure 2). FIRs and FBW7 expression was relatively constant in these cell lines regardless of TP53 mutation status. Remarkably, Notch 1 was increased in pathogenic TP53 cells, YES2 and T.Tn, than those of TP53-benign cells (Figure 1D, small arrows). Note, the TP53 gene status was not consistently affected cyclin E, FIRs, FBW7 and SAP155 expression in ESCC cells (Figure 1C). Together, no apparent feedback reaction was observed in FBW7 expression in response to altered TP53 or Notch1 expression in ESCC cells (Figure 1D). Therefore, the reason for increased TP53 expression in ESCC tissues was likely due to decreased FBW7 expression rather than the stabilization by p53 gene mutation. Cyclin E and FBW7 expressions were both high in some cancers where FIRs were also highly expressed (Figure 1D) indicated by immune-histochemical staining.

### FIRs, authentic FIR and AS form of FIR (FIRΔexon2), were overexpressed in ESCC tissues

**Figure 2:**
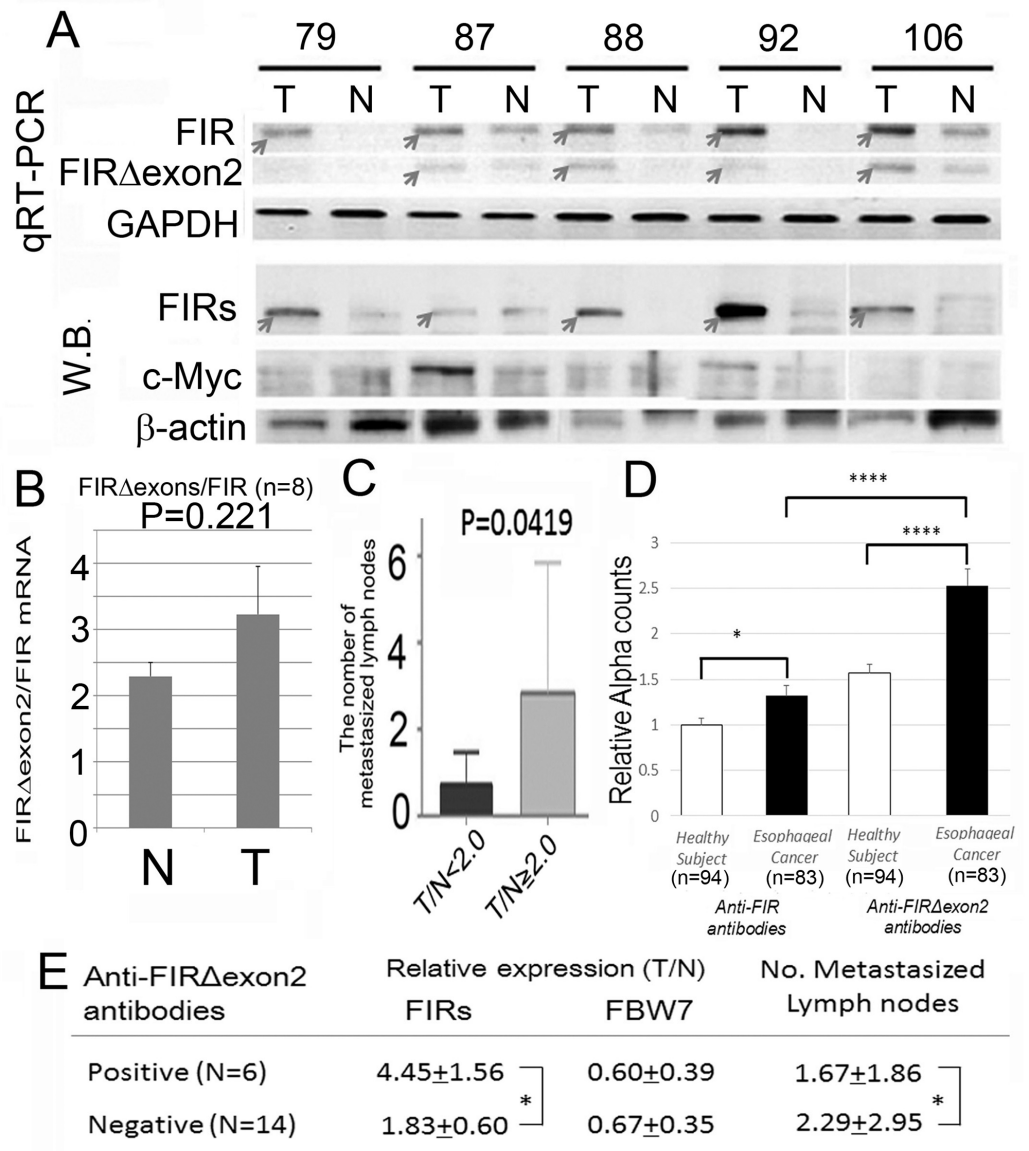
Alternatively spliced form of FIR (FIRΔexon2) was expressed in ESCC tissues and anti-FIRΔexon2 autoantibodies were detected in the serum of the patients **(A)** The expression levels of FIRΔexon2 and FIR mRNA in esophageal cancer (T) and corresponding non-cancer tissues (N) were examined by qRT-PCR and Western blot. Both FIR and FIRΔexon2 mRNAs were higher in cancer tissue than in non-cancer tissues. **(B)** The ratio of FIRΔexon2/FIR mRNA expression was higher in esophageal cancer tissues (T: 3.23 in average) than in non-cancer tissues (N: 2.29 in average) without statistical significance (*t*-test, P=0.221). Note, c-Myc expression showed no significant difference between esophageal cancer and non-cancer tissue examined in this experiment. **(C)** The degree of expression of FIRs was correlated positively with the number of metastasized lymph nodes. In serum of esophageal cancer patients, anti-FIRΔexon2 autoantibodies were detected compared to FIR. **(D)** Relative anti-FIR or anti-FIRΔexon2 antibodies detected in the sera of esophageal cancer patients. **(E)** Anti-FIRΔexon2 autoantibodies, FIRs, and FBW7 expression in cancer tissues. The average of the number of lymph nodes metastasis was significantly smaller in positive cases (N=6, 1.67+1.86) than in negative cases (N=14, 2.29+2.95) in terms of anti-FIRΔexon2 autoantibodies detection. P-values were calculated by Student’s *t*-test. ^*^P<0.05, ^****^P<0.0001. Error bars indicated standard error. Esophageal cancer tissue (T) and non-cancer tissues (N) were examined without chemoradiation therapy before operation.

The AS form of FIR has been reported in cancers with high levels of c-Myc, such as in colorectal cancer, [10] hepatocellular carcinoma, [13] nonsmall lung cancer, [14] and T-cell acute lymphoblastic leukemia [21]. In this study, FIRΔexon2, at the mRNA and protein levels, was elevated in ESCC (Figure 2A, upper panels). FIRΔexon2 activates *c-myc* gene transcription *in vitro* through a possibly dominant negative effect of FIR [10]. Even though FIRΔexon2 was elevated in ESCC, c-Myc was not activated (Figure 2A, lower panels). FIRΔexon2/FIR mRNA was significantly elevated in ESCC tissues (Figure 2B). These results indicated that a splicing form of FIR, FIRΔexon2, expressed and existed for a certain period of time in esophageal cancer tissues. Clinically, esophageal cancer patients with high FIRΔexon2/FIR ratio (tumor tissues/non-tumor tissues [T/N] ratio) of ≥ 2 had significantly higher lymph node metastases (Figure 2C).

### Anti-FIRΔexon2 autoantibodies were detected in the serum of ESCC patients

Moreover, anti-FIRΔexon2 autoantibody (IgG) was detected in other gastrointestinal cancers, [23, 24] and anti-FIRΔexon2 autoantibody was examined in serum of esophageal cancer patients in this study (Figure 2D). Interestingly, anti-FIRΔexon2 autoantibodies were detected in sera of esophageal cancer patients who had high FIRs expression in cancer tissues with significantly decreased lymph nodes metastasis. Therefore, antibodies against FIRΔexon2 can be used as a potential biomarker for esophageal cancer and a therapeutic target to reduce the lymph nodes metastasis (Figure 2E). Clinical features of the esophageal cancer patients and the results of relative expression of FBW7 (T/N), FIR (T/N), and the number of metastasized lymph nodes are listed (Table 1).

**Table 1:** The clinical features of the patients are summarized

No. cases	Gender	Age	Stages	Pathology	Anti-FIRΔexon2 Abs	FIRs (T/N)	FBW7 (T/N)	No.meta LNs	No. LNs examied
1	Male	72	Stage 0	mod.diff	positive	5.1	0.5	0	72
2	Male	73	Stage I	por.diff	positive	4	0.2	0	69
3	Male	77	Stage I	mod.diff	positive	6.9	1.1	3	50
4	Male	74	Stage III	mod.diff	positive	3.7	0.3	4	119
5	Male	69	Stage II	mod.diff	positive	5	0.5	3	45
6	Female	70	Stage III	mod.diff	positive	2.3	1.1	0	32
7	Male	80	Stage III	mod.diff	positive	2.9	0.9	0	28
8	Male	57	Stage II	mod.diff	negative	1.3	0.9	0	68
9	female	52	Stage III	por.diff	negative	2.4	1.1	9	35
10	Male	77	Stage II	wel.diff	negative	1.2	0.2	1	107
11	Male	61	Stage III	wel.diff	negative	1.7	1	0	89
12	Female	74	Stage II	mod.diff	negative	1.5	1.1	1	51
13	Female	83	Stage I	wel.diff	negative	1	0.6	1	39
14	Male	79	Stage IVa	basaloid SCC	negative	2.1	0.3	5	85
15	Male	66	Stage II	mod.diff	negative	2.2	0.8	2	57
16	Male	69	Stage II	mod.diff	negative	1.1	0.8	2	83
17	Male	65	Stage III	mod.diff	negative	2	0.7	0	63
18	Male	74	Stage II	mod.diff	negative	2.1	0.2	8	57
19	Female	70	Stage III	mod.diff	negative	2.5	0.2	1	83
20	Male	82	Stage II	por.diff	negative	N.D.	0.2	2	46
21	Male	65	CRT	mod.diff	negative			0	76
22	Male	67	CRT	Grade2	negative			0	34
23	Male	58	CRT	mod.diff	negative			3	66
24	Male	60	CRT	mod.diff	negative			1	71
25	Female	55	CRT	Grade2	negative			1	46

Abbreviations: CRT: chemo-radiation-therapy, wel.diff: well differentiated ESCC, mod.diff: moderately differentiated ESCC, por.diff: poorly differentiated ESCC.

### Knockdown of FIR splicing variant, FIRΔexon2, by siRNA reduced cyclin E in ESCC cells under even benign TP53 status

**Figure 3:**
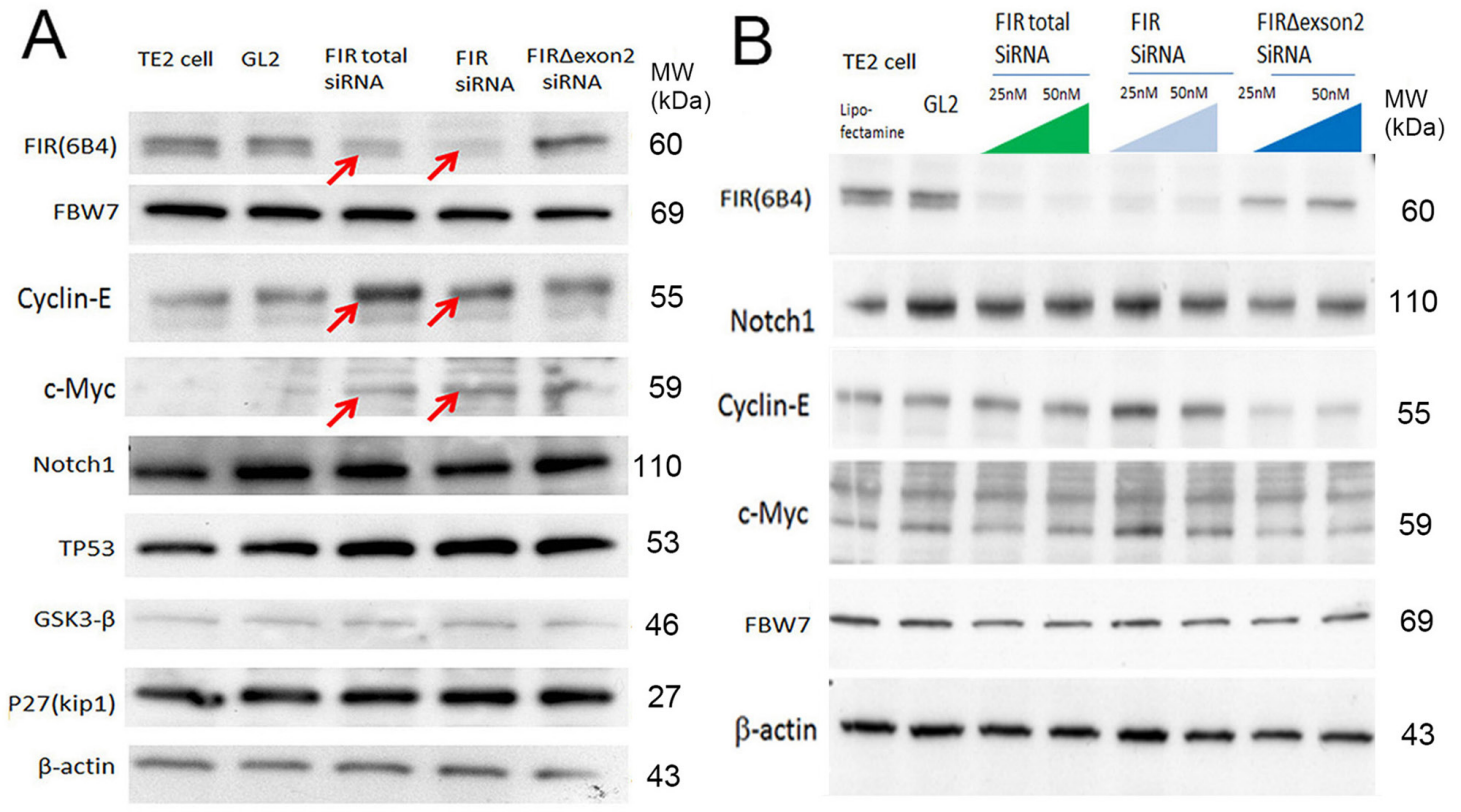
FIRΔexon2 was required for cyclin E expression in ESCC cells **(A)** The effect byFIRs, FIR andΔexon2 siRNA to FBW7, cyclin E, c-Myc, Notch1, TP53, GSK3-β and P27(Kip1) were examined in TE2 cells (benign TP53). **(B)** Total FIR and FIR siRNA increased cyclin E in ESCC cells (TE2 cells) in a dose dependent manner. FIRΔexon2 siRNA suppressed cyclin E expression in ESCC cells.

If the expression of FIRs is critical for cyclin E expression, altered FIRs expression should alter its expression. To explore the significance of FIRs, FIR, and FIRΔexon2 expression in relation to esophageal cancer progression, knockdown of FIR was challenged to esophageal cancer cells TE2 (TP53 gene variation was benign) and cyclin E expression was examined. As expected, FIR siRNA increased cyclin E in esophageal cancer cells, TE2 (Figure 3A, arrows and 3B). The expression of TP53 and GSK3β were not affected by siRNA of FIRs, FIR, and FIRΔexon2 (Figures 3A). Further, FIRΔexon2 siRNA decreased cyclin E expression in TE2 indicated FIRΔexon2 is required for sustained cyclin E expression (Figures 3B).

On the other hand, the direct or indirect interaction between FIR and SAP155 sustains the mutual proteins expression [15]. To be precise, FIR forms a complex with SAP155 (SF3b1), that is the carboxyl-terminus of FIR/PUF60 directly interacts with SAP155 (SF3b1) [25] and SAP155 is required for FIR protein expression and vice versa [11, 15]. Therefore, knockdown of SAP155 was also expected to decrease cyclin E and Notch1.

### Knockdown of SAP155 (SF3b1) by siRNA reduced cyclin E and Notch1 depend on the TP53 expression level in ESCC cells

**Figure 4:**
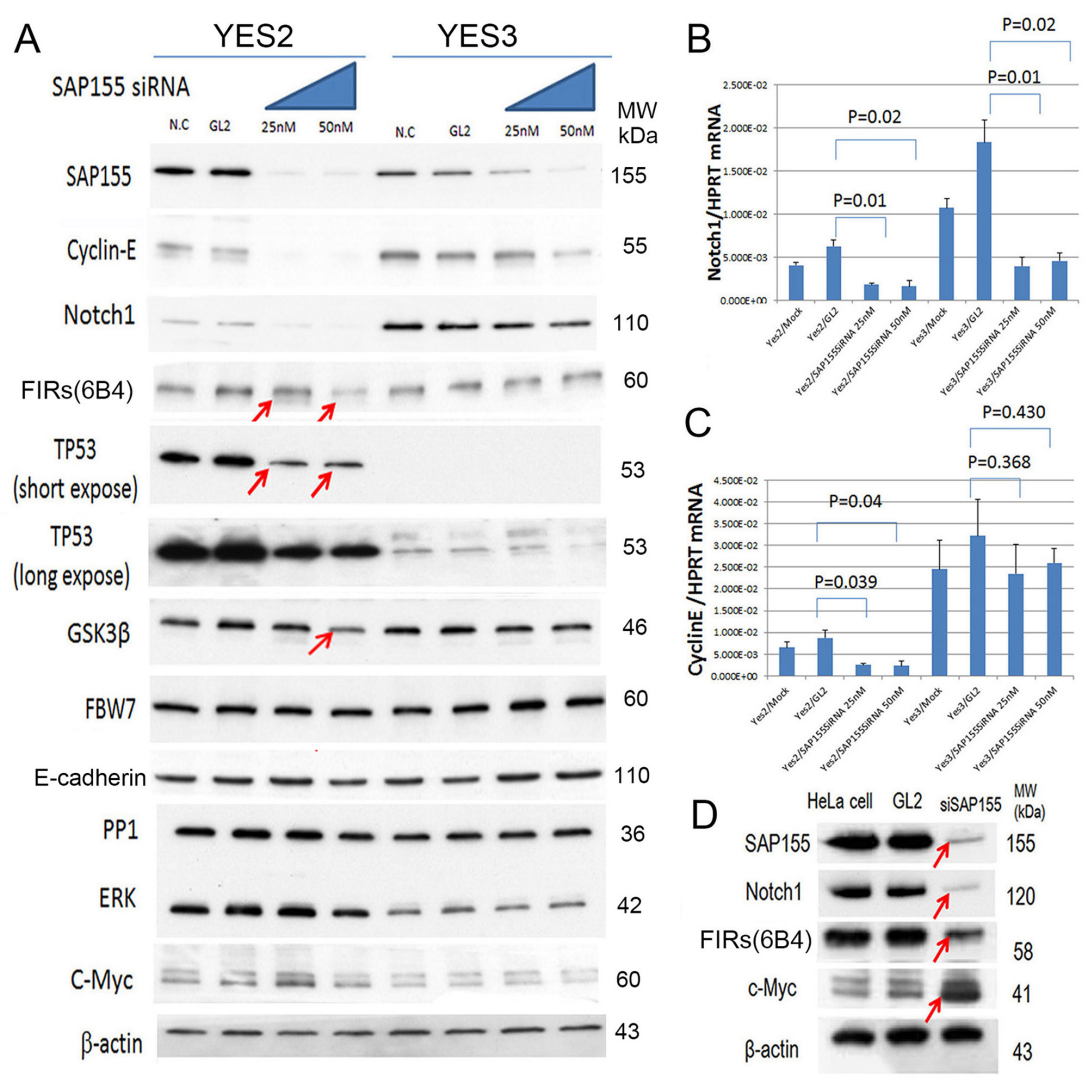
SAP155 (SF3b1) was required for cyclin E and Notch 1 mRNA expression in ESCC cells SAP155 (SF3b1) siRNA suppressed cyclin E, TP53, Notch 1, FIRs, TP53, GSK3β, FBW7, E-cadherin, PP1, ERK and c-Myc expression in esophageal cancer cells, YES2 and YES3. **(A)** SAP155 siRNA decreased cyclin E, Notch1 FIRs and TP53 expression in YES2 (pathogenic TP53), but observed less effective in YES3 cells (truncated TP53 expression). FBW7 expression was irrelevant to SAP155 siRNA in YES2 and YES3 cells. Note, GSK3-β was suppressed by SAP155 siRNA in YES2 cells. FBW7, E-cadherin, PP1, ERK and c-Myc were not affected by SAP155 siRNA in YES2 and YES3 cells. **(B)** qRT-PCR of Notch1 mRNAby SAP155 siRNAin YES2 and YES3. **(C)** qRT-PCR of cyclin E mRNA by SAP155 siRNAin YES2 and YES3. **(D)** SAP155 siRNA decreased Notch1 and TP53 expression in HeLa cells (benign TP53).

Since cyclin E was reduced by FIR siRNA, the effect of SAP155 siRNA to cyclin E was examined in the ESCC cells with different endogenous TP53 expression level. Remarkably, the knockdown of SAP155 by siRNA drastically decreased cyclin E, Notch1, FIR, TP53 and GSK3β in YES2 cells with pathogenic TP53 (Figure 4A, arrows) whereas those no remarkable changes were observed in YES3 cells with scarce TP53 expression (Figure 4A, arrows). Of note, the knockdown of SAP155 by siRNA significantly suppressed Notch1 mRNA (Figure 4B) but not cyclin E mRNA (Figure 4C). These results indicated that TP53 and SAP155 expression is critical for Notch and cyclin E expression at their mRNA and protein level (Figure 4). SAP155 siRNA suppressed FIRs in HeLa (benign TP53) cells (Figure 4D). These results suggested that suppression of cyclin E by SAP155 siRNA could be pursued via a post-transcriptional mechanism. Incidentally, Notch1, cyclin E, and TP53 are the substrates of FBW7. Recently, FBW7 has been identified as a direct *bona-fide* transcriptional target of TP53 and adenoviral TP53 increased proteosomal degradation of c-Myc and cyclin E [26, 27]. FBW7 expression was irrelevant to TP53 expression in YES2 and YES3 in this study. Further, the effect of knockdown of SAP155 to cyclin E and Notch1 was not significant in YES3 (Figure 4A) and knockdown of FIRΔexon2 suppressed cyclin E in TE2 (Figure 3B), indicating the presence of FIR/FIRΔexon2 protein expression disturbed FBW-related post-transcriptional regulation of cyclin E and Notch1 independent on TP53 expression. Since FBW7 is suppressed whereas TP53 and cyclin E expression was increased in ESCC tissues (Figure 1), one possible mechanism is that PUF60/FIR/FIRΔexon2 potentially interferes with FBW7 and inhibits proteolysis of cyclin E.

### Three-dimensional structure analysis of hypothetical inhibitory mechanism of FBW7 protein by FIR/FIRΔexon2

**Figure 5:**
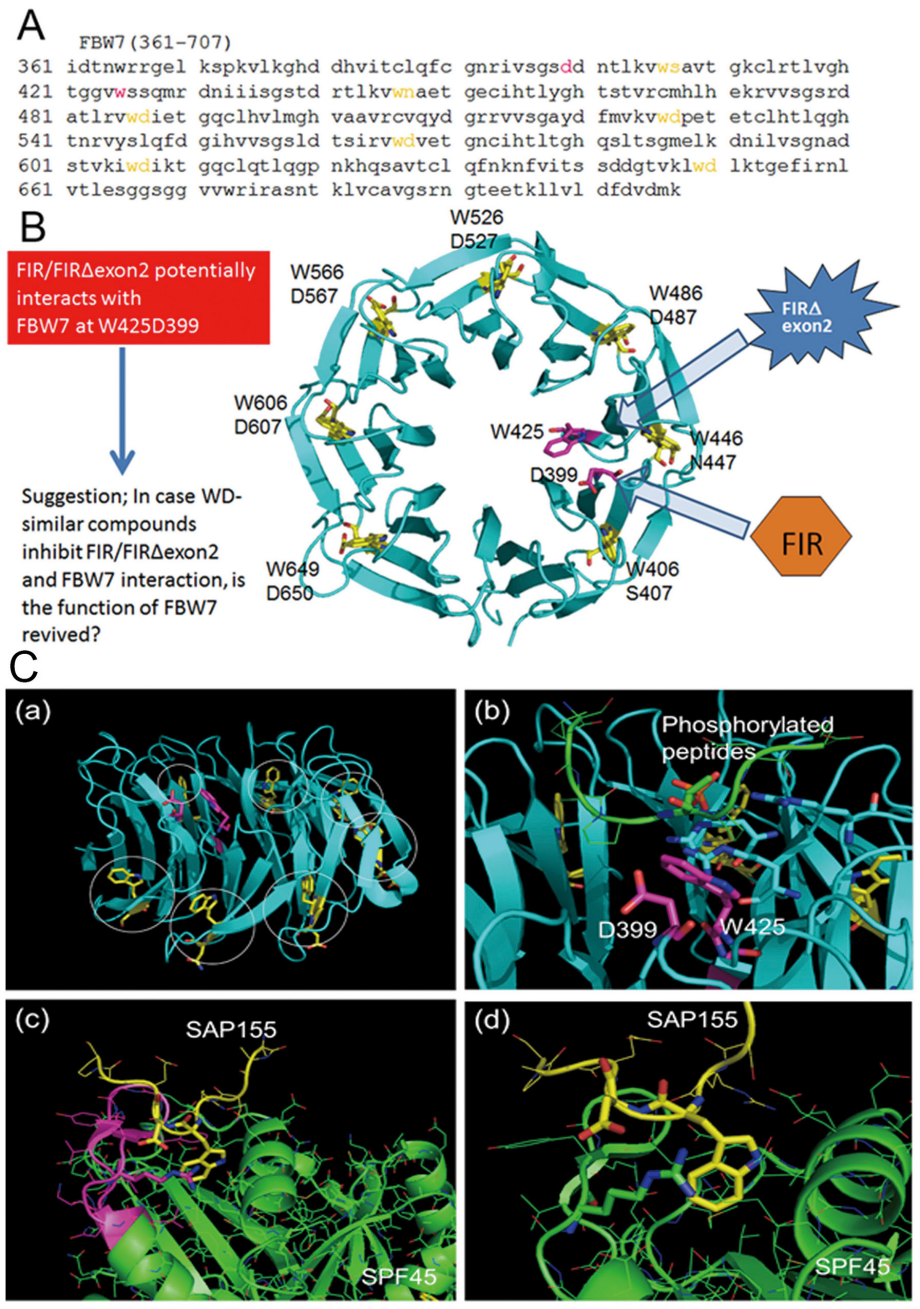
Potential interaction between FBW7 and FIR/FIRΔexon2 revealed by computational three-dimensional crystal structural analysis Three-dimensional crystal structural analysis of FBW7 revealed that Trp (W) and Asp (D) were located next to each other in its degron pocket as a WD domain-like structure that possibly bound to the UHM-domain in the carboxyl-terminus of FIR. **(A)** Amino acids sequence (aa. 361–707) of substrate binding site is indicated. CPD bond propeller pocket in FBW7 also is known as the “degron pocket.” Neighboring W and D (indicated in *yellow*) potentially binds to FIR-UHM (see text). **(B)** All neighboring WD domain (indicated in *yellow*) formed the structural backbone of the “degron pocket” of FBW7. Interestingly, WD-like motifs (W425 and D399) of the “degron pocket” are closely located to each other in 3D structure after protein folding, and it has been suggested that they interact with FIR-UHM (LNGRWFAGRKVVA) (indicated in *magenta*). **(C-a)** Structure of CPD of FBW7. WD motifs to stabilize the CPD folding are colored *yellow* and indicated by *circles*. W and D residues colored *magenta* are not involved in the CPD folding. **(C-b)** Binding of a phosphorylated peptide to CPD. The phosphate group makes an interaction with three R residues shown by the *stick* representation. The W and D residues not involved in the CPD folding are positioned at the middle of CPD. **(C-c)** Interaction of SAP155 and SPF45. The LNGRYFGGRVVKA motif of SPF45 is colored *magenta*. **(C-d)** Close view of the SAP155-SPF45 interaction. W and D residues of SAP155 shown in *yellow* made a strong interaction with R304 of SPF45, depicted in the *stick* representation.

Three-dimensional structure analysis revealed that FIRΔexon2 potentially interacts with FBW7 directly and inhibits c-Myc degradation. C-terminal domain of FIR belongs to the family of U2 auxiliary factor (U2AF) homology motifs (UHM), a subgroup of RNA recognition motifs (Figures 5A, 5B). FIR-UHM has distinct binding preferences to the WD domain in N-terminus of SAP155 [25]. FBW7 is a polyubiquitin ligase that acts on proteins, such as c-Myc and Notch1, which include consensus sequence of Cdc4 phospho-degron (CPD; --TP S/E--). Therefore, FIRΔexon2 potentially interacts directly with FBW7 and inhibits the function of poly-ubiquitination of FBW7. FBW7 usually recognizes phosphorylated peptides with the amino sequences of --TP---S-- or --TP---E--. According to the crystal structure of the complex of FBW7 β-propeller domain and a phosphorylated peptide (PDB#: 2QVR), phosphate groups of the peptide made a strong electrostatic interaction with three basic polar residues (Arg), and the peptide is held firmly on the center of FBW7 β-propeller domain. The β-propeller domain has seven times repeats of the WD motif. These WD repeats are involved in the folding and stability of the domain and then hardly related to the recognition of phosphorylated peptides (Figure 5C-a). It is interesting to note that another combination of Trp (W) and Asp (D) exists on the FBW7 β-propeller domain that is not involved in the folding. These W and D residues are slightly apart from each other in the amino acid (aa.) sequence (W425 and D399), but they both are positioned at the middle of the β-propeller domain and are close to each other (Figure 5C-b). FIR has an amino acids sequence of LNGRWFAGRKVVA at its C-terminal side (aa. 505–517). An mRNA splicing factor 45, SPF45, has an almost same sequence of LNGRYFGGRVVKA at its RNA-binding site (aa. 301–401). SPF45 was reported to be bound to SAP155 through this motif [28]. A crystal structure on the binding mode of SPF45 and SAP155 (PDB#: 2PEH) suggested that SAP155 used W and D residues for the binding and W338 and D339 of SAP155 made a close contact with the SPF45 LNGRYFGGRVVKA motif (Figures 5C-c, 5C-d). SFP45 is an mRNA splicing factor and has a common sequence to FIR at its RNA-binding site. Two crystal structures, 2QVR and 2PEH, were visualized by PyMOL [DeLano, W. L.; The PyMOL Molecular Graphics System, Schrödinger, LLC]. A comparison of two crystal structures indicates that the positions and configurations of W and D in FBW7 are considerably similar to those of SAP155. Hence, FBW7 is possibly bound to FIR in the analogy to SAP155 to SPF45. A complex structure of FBW7 and its substrate peptide was observed in 2QVR and a binding structure of SPF45 and SAP155. Namely, the WD-like motifs of the β-propeller pocket in FBW7 are located closely to each other in 3D structure after protein folding, which enables the β-propeller pocket to interact with FIR-UHM (LNGRWFAGRKVVA; Figure 5B). Therefore, it is suggested that FIRΔexon2 can maintain the c-Myc protein levels after transcription by inhibiting the FBW7 pathway (Figure 5).

### *In vitro* binding affinity between FIRΔexon2 and FBW7

The titration curve of FBW7 with FIR Δexon2 suggested the molecular interaction between two proteins (Supplementary Figure 3). The exothermic peaks were observed in the initial 17 injections (Supplementary Figure 3, range A). The peak level was decreased in the later injections (Supplementary Figure 3, range B). Due to the sudden change in ITC thermogram, the binding affinity cannot be determined reliably, but it will be over 10^8^ M^-1^. Since the binding reaction is exothermic, the binding of FBW7 and FIR Δexon2 is enthalpically driven. This result is compatible with the importance of Asp of FBW7 in the molecular binding, because Asp usually contributes to hydrophilic interaction such as hydrogen bond formation between protein molecules.

### DNA damaging agents, CDDP or 5FU, increased cyclin E expression in ESCC cells

**Figure 6:**
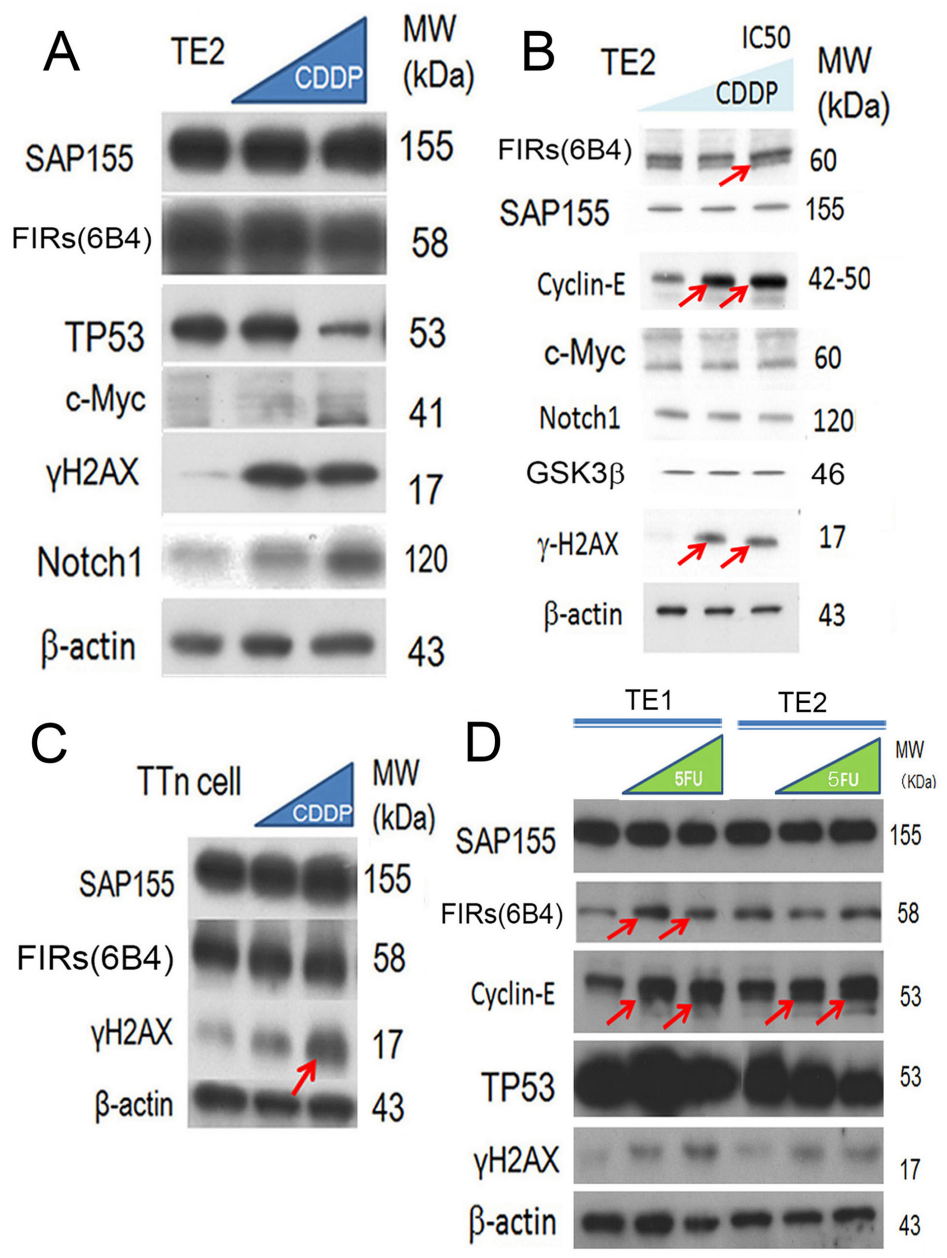
CDDP (cisplatin) treatment reduced the expression of TP53 and FIR in ESCC cells **(A)** Esophageal cancer cell lines (TE2) were exposed to CDDP. The expression of TP53, and FIR was decreased, whereas that of Notch1 was increased. **(B)** Cyclin E was increased by CDDP treatment (*arrows*). **(C)** γH2AX was an indicator of DNA damage as well as T.Tn cells that has pathogenic TP53 mutation (arrows). **(D)** 5FUtreatment increased cyclin E expression in esophageal cancer cells (TE1 and TE2 cells).

DNA damage by 5-FU has been reported to affect FIR, P27Kip1/cyclin E, and Ku86/XRCC5 expression [4]. The findings in this study indicated that cyclin E overexpression rather than c-Myc is responsible for the proliferation or progression in ESCC. When cancer cells are exposed to bleomycin (BLM), altered FIR splicing is induced with decreased expression of SAP155 [4]. In this study, we investigated whether the expression of cyclin E is affected by DNA-damaging anticancer drugs, CDDP or 5FU, in ESCC TE2 cells [29] (benign TP53 variation). Expectedly, TP53 was decreased (Figure 6A, arrows), whereas cyclin E was increased (Figures 6B, 6C, arrows) when exposed to CDDP in esophageal cancer cell lines (TE2 and TE1 with benign TP53 variation). γH2AX was an indicator of DNA damage even in T.Tn cells that has pathogenic TP53 mutation ((Figure 6C, arrow). Previously, FIR delayed DNA damage repair in hepatoblastoma cells [4] and adenovirus-mediated FIR overexpression enhanced DNA damage caused by carbon ion particle beams in a xenograft-esophageal carcinoma mouse model [30]. Therefore, the expression of the FIRs is required for the CDDP-resistance mechanism. FIRs expression was relatively constant under CDDP or 5FU treatment in esophageal cancer cells T.Tn (pathologic TP53) and TE1/TE2 (benign TP53) (Figures 6C, 6D).

## DISCUSSION

**Figure 7:**
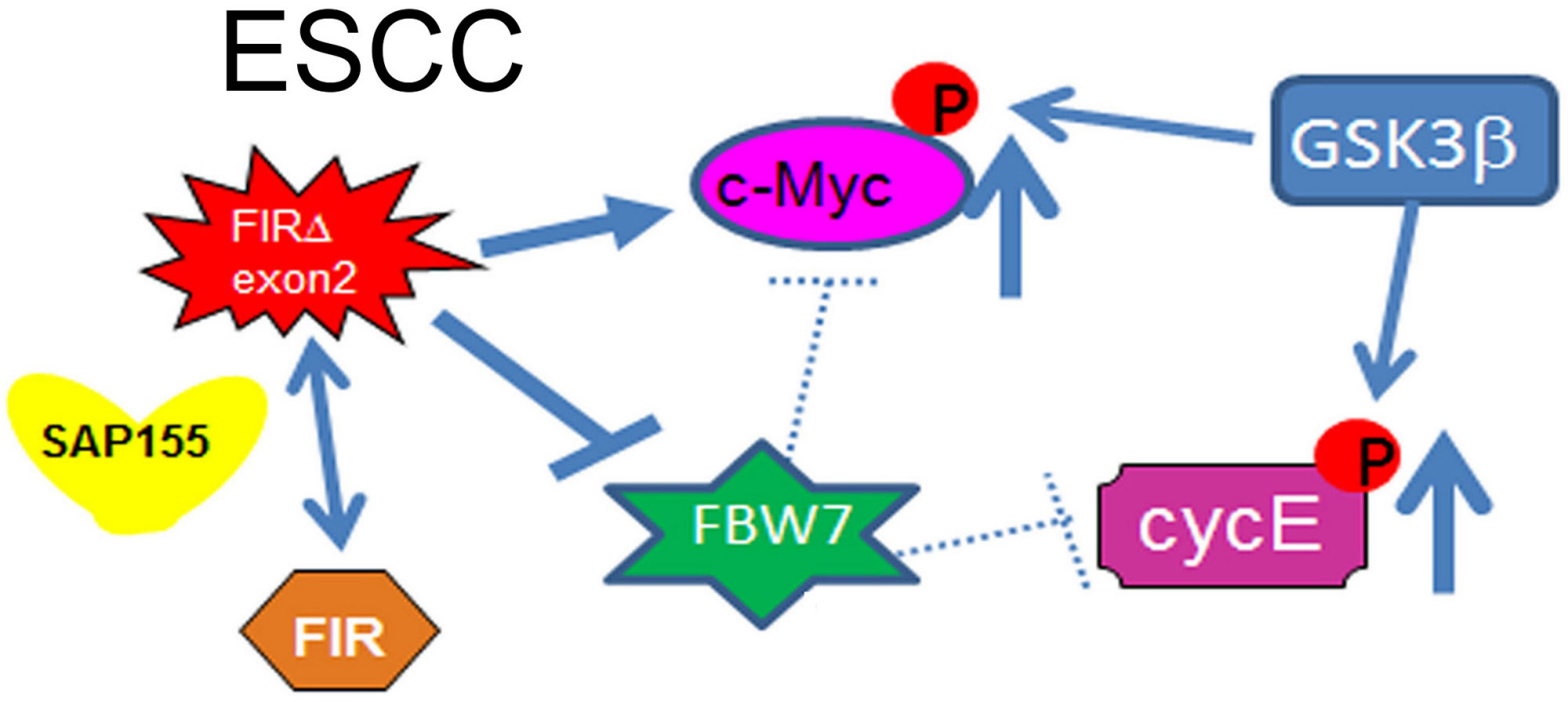
Cyclin E expression in ESCC in terms of the potential interaction between FIR/FIRΔexon2 and FBW7 The extent of mRNA suppression of Notch1 and cyclin E by SAP155 siRNA was different depending on the TP53 expression in ESCC cells. Since SAP155 is required for proper alternative splicing of FIR (PUF60) pre-mRNA, knockdown of SAP155 recovered FBW7 function possibly by reducing FIR/FIRΔexon2 expression. Therefore, the function rather than the expression of FBW7 was obstructed directly or indirectly by reduced SAP155 expression. The AS form of FIR, FIRΔexon2, inhibits FBW7 function in esophageal cancer cells. Note, the knockdown of SAP155 (SF3b1) expression induced the decrease of Notch1 and cyclin E, which are substrates of FBW7. SAP155 siRNA rescued FBW7 function by potentially reducing FIR/FIRΔexon2 expression. Three-dimensional structure analysis revealed that a WD-like motif exists on the degron pocket of FBW7 protein that potentially interacts with FIR/FIRΔexon2 (see text).

Concerted linkage of FBW7, ERK and GSK-3β regulates cell cycle and stem cell maintenance through post-transcriptional expression of c-Myc and cyclin E [31, 32]. This study proposed a novel mechanism that AS of FIRs expression is strongly related to cyclin E overexpression by inhibiting the degron pocket of FBW7 which is pivotal for proliferation in ESCC. The increased expression of the AS form of FIR (FIRΔexon2) and cyclin E, and decreased FBW7 expression were observed in ESCC compared to normal tissues (Figures 1A, 1B). Cyclin E and Notch1 expression were different among TP53 mutational status (Figure 1C). FIRΔexon2 existed at mRNA and protein levels in ESCC tissues because anti-FIRΔexon2 autoantibodies were detected in the sera of those patients (Figure 2). Antibodies against FIRΔexon2 are potential biomarker for esophageal cancer and a therapeutic target to decrease lymph nodes metastasis (Figures 2D, 2E). Moreover, knockdown of FIR by siRNA increased cyclin E expression in esophageal cancer cells (Figure 3, arrows). Knockdown of SAP155 (SF3b1) by siRNA decreased cyclin E, Notch1, TP53 expression (Figure 4A) and FIRs as expected [15]. The extent of mRNA suppression of Notch1 and cyclin E by SAP155 siRNA was different depending on the TP53 expression when compared YES2 with YES3 cells (Figures 4A, 4B). These results indicated that the function rather than the expression of FBW7 was obstructed directly or indirectly by reduced SAP155 expression. Since SAP155 is required for proper AS of FIR (PUF60) pre-mRNA, [11] knockdown of SAP155 recovered FBW7 function possibly by reducing FIR/FIRΔexon2 expression. Three-dimensional structure analysis revealed that a WD-like motif exists on the degron pocket of FBW7 protein that potentially interacts with FIR/FIRΔexon2 (Figure 5). DNA-damaging agents, CDDP or 5FU, increased cyclin E expression in ESCC TE2 cells [29] (Figure 6). These results indicated that the expression of FIR/FIRΔexon2 affects degradation of cyclin E and Notch1 by FBW7 depending on the TP53 (Figure 7A). Knockdown of SAP155 by siRNA reduced FIR/FIRΔexon2 and TP53 expression with sustained FBW7 expression (Figure 7B). Together, PUF60/FIR/FIRΔexon2 potentially interferes with the degron pocket of FBW7 from cyclin E degradation (Figure 7). In summary, FBW7 expression was significantly decreased, whereas cyclin E was significantly increased in excised human ESCC tissues. CDDP-induced DNA damage increased Notch1 and decreased FIR expression with altered FIR splicing, which potentially enhances cyclin E expression. The process from DNA damage to cell proliferation was indicated via c-Myc–independent cyclin E in esophageal cancer. Even though p53 is mutant, it’s stability/expression was suppressed by SAP155 (Figure 4). In case the change of mutant p53 proteins expression, it does not directly represent or surrogate the effect of RNA interference of FIR and SAP155 and the DNA-damaging agents. To examine the protein expression level depends on FBW7-mediated ubiquitin system in ESCC cells, proteasome inhibitor need to be tested. Further study is required to reveal a more detailed mechanism.

When ESCC cells (TE2) and cervical SCC cells (HeLa cells) were exposed to anticancer agents (CDDP and 5-FU), expression of FIR was reduced. FBW7 was not expressed in esophageal cancer cell lines, but it has been reported that FBW7 has high frequency variation in colorectal cancer [33] and FBW7 upregulation enhanced CDDP cytotoxicity in nonsmall cell lung cancer cells [24]. FIR was co-immunoprecipitated with Ku86 and DNA-PKcs. siRNA against Ku86/Ku70 decreased FIR and P27Kip1 expression, whereas siRNA against FIR decreased Ku86/XRCC5 and P27Kip1 expression. Accordingly, the interaction of FIR/FIRΔexon2 bridges c-Myc and P27Kip1 expression [4]. Fascinatingly, *FIR* haplodeficiency promotes splicing to pyruvate kinase M2 in mice thymic lymphoma tissues, indicating disturbed splicing of FIR or a dominant negative form of FIR interferes cancer metabolism [34]. Adenovirus vector–mediated FIR expressions enhanced the efficacy of heavy particle beam on the subcutaneously xenografted esophageal cancer cells into the thigh of nude mice (BALB/c), indicating increased DNA damage or delayed DNA damage repair [22]. Together, FIR is involved in cancer development and DNA damage repair resistance. In other words, altered FIR expression inhibits FBW7, resulting delayed DNA damage repair or elevated cyclin E expression in a c-Myc–independent manner in esophageal carcinogenesis. Previous studies showed the correlation of p53 and tumor markers in pancreatic adenocarcinoma; p53 influenced lymph metastasis in colorectal cancer [35, 36].

Since FBW7 ubiquitinates c-Myc and Notch1 proteins and promotes degradation of those proteins in the proteasome system, a mutation in the FBW7 gene increases intracellular accumulation of c-Myc and Notch1 proteins [20, 37]. This study revealed that increased cyclin E with decreased FBW7 expression could be crucial for proliferation in ESCC. Isothermal titration calorimetry (ITC) revealed a direct or indirect interaction between FWB7 and FIRΔexon2 (Supplementary Figure 3). However, FWB7 was not confirmed by pull-down assay with FLAG-beads with nuclear proteins of FIRΔexon2-FLAG expressed HeLa cells [12]. An undetermined tertiary factor is potentially required for the interaction between FBW7 and FIR, such as JAZ repression of MYC in jasmonate signaling [38]. Together, small molecules or antibodies (scFv) that inhibit FIRΔexon2 are candidates to suppress tumor metastasis and revealing the effects of FIR/FIRΔexon2/SAP155 complex to FBW7-related proteolysis are promising targets for future diagnostic and therapeutic applications.

## MATERIALS AND METHODS

### Human samples, cells, and reagents

Human ESCC tissues from 25 patients were obtained at tumor resections in the Department of General Surgery, Chiba University Hospital, Chiba, Japan. A total of 20 patients with esophageal cancer had no treatment before operation and proteins were extracted for further Western blot analysis (Table 1). Definition of stages of ESCC in this study was categorized according to Japanese classification of esophageal cancer (Supplementary Table 1). This study was conducted in accordance with “The Code of Ethics of the World Medical Association” (Declaration of Helsinki). This work was approved by the Local Ethical Review Board of the Chiba University, Graduate School of Medicine, and those of co-operating hospitals. Sera of patients with ESCC (n=83) were obtained at the Department of Frontier Surgery, Chiba University Hospital, Chiba, Japan. Sera of health donors (HDs) (n=94; female 40, male 54: 54.8 + 9.1 years old) were obtained from Higashi Funabashi Hospital, Chiba Japan [23, 24]. Written informed consent was obtained from each patient before surgery approved by ethical committee of Graduate School of medicine, Chiba university. The sera were extracted and stored at −80°C until analysis for detecting anti-FIRs autoantibodies from an additional 83 esophageal cancer patients. The laboratory data were obtained before operation. All excised tissues were placed immediately in liquid nitrogen and stored at −80°C until analysis. The human ESCC cell lines, T.Tn, TE1, TE2, YES2, and YES3, were obtained from the Japan Cell Research Bank. Human cervical SCCs (HeLa cells) were purchased from the American Type Culture Collection (ATCC). All cell lines were cultured in Dulbecco’s modified Eagle medium (DMEM) supplemented with 10% fetal calf serum (FBS; Invitrogen, Tokyo, Japan) and 1% penicillin-streptomycin, and they were cultured at 37°C in a humidified atmosphere containing 5% CO_2_.

### Protein extraction

Frozen tissue was pulverized and 25 mg of tissue was mixed with VER7 (solvent). The mix was homogenized three times with Polytron (Thermo Fisher Scientific Inc., MA, USA) for 30 s–1min per homogenization. The homogenate then was centrifuged using an ultra-high speed centrifuge for 1 h at 50K. The supernatant was collected and stored at −80°C.

### Western blotting and antibodies

ESCC tissues were obtained from 20 patients who had received no preoperative radiotherapy or chemotherapy. In case cancer cells, culture medium was removed and the cells were washed twice with cold (4°C) phosphate-buffered saline (PBS), lysed with 1:20 β-mercaptoethanol and ×2 sample buffer, and incubated at 100°C for 5 min. Whole-cell lysates were assayed for protein contain (Bio-Rad, Hercules, CA, USA), and 10 μg of proteins were separated by sodium dodecyl sulfate-polyacrylamide gel electrophoresis (SDS-PAGE) on 10% to 20% XV PANTERA gels and transferred onto polyvinylidene fluoride membranes using a tank transfer apparatus. The membranes were blocked with 0.5% skim milk in PBS for 1 h in room temperature or overnight at 4°C. Membranes were incubated with primary antibodies for 1 h at room temperature, followed by three 10-min washes with 1 × PBS/0.01% Tween 20. Membranes then were incubated with commercial secondary antibodies, followed by three 15-min washes with 1 × PBS/0.01% Tween 20. The primary mouse monoclonal antibody against FIR C-terminus (Total FIRs 6B4) was prepared by Dr Nozaki [11]. Details of other antibodies used in this study are listed (Supplementary Table 2).

### Quantitative reverse transcription polymerase chain reaction (qRT-PCR)

Total RNA was extracted from HeLa cells using the RNeasy Mini Kit (Qiagen). cDNA was synthesized from total RNA by the first strand cDNA Synthesis Kit for RT-PCR (Roche). Using the cDNA as a template, FIR cDNA was amplified with suitable primers by RT-PCR (Supplementary Table 3, Supplementary Figure 1). Glyceraldehyde-3-phosphate dehydrogenase cDNA was amplified and used as the control. The PCR product was loaded on a 2.5% agarose gel (Promega), purified with the Gel Extraction Kit (Qiagen), and cloned using the pGEM-T Easy vector system (Promega) for DNA sequencing. The positions of forward primers for qRT-PCR amplification of FIR and FIRΔexon2 (Supplementary Table 1) were described previously [12] and briefly illustrated (Supplementary Figure 1).

### Detection of *TP53* gene mutations in ESCC cells

The procedures for *TP53* gene mutation detection analysis of cancer cell lines were described previously [10, 23]. The specific primers and PCR conditions were indicated (Supplementary Table 3). The significance of ***TP53*** gene mutation or variation status was evaluated by COSMIC (catalogue of somatic mutations in cancer: http://cancer.sanger.ac.uk/cosmic) data base.

### Purified FIRΔexon2 and FBW7 proteins

*FIR*Δ*exon2* cDNA [10] was inserted into pET-50b (+) DNA plasmid vector. An *Escherichia coli* strain, Rosetta (DE3) pLysS (competent cells), that was transformed with the pET-50b-FIRΔexon2 vector, were cultured in 4L Luria-Bertani (LB) medium at 30°C until the OD600 value reached 0.5–0.6, followed by 12 h additional culture with an addition of isopropyl β-D-1 thiogalactopyranoside (IPTG; inducer of protein expression). A cell pellet obtained with centrifugation of the cultured medium was resuspended in a buffer containing 10mM imidazole and 1mM phenylmethylsulfonyl fluoride (PMSF). After disrupting bacterial cell membrane by sonication, the protein was firstly purified by a Ni-affinity column with a gradient rise of the imidazole concentration up to 500 mM. The eluted protein fraction was dialyzed overnight against the buffer without imidazole. The Nus-tag was cleaved by HRV-3C protease and the protein was secondly purified by a Co affinity column to remove the cleaved Nus-tag. The protein was thirdly purified by an anion exchange column with a gradient increase of NaCl concentration. The protein was finally purified by a gel filtration with a running buffer of 10 mM Tris-HCl at pH 8.0 and 150 mM NaCl. FBW7 was expressed as a complex with Skp-1, using pCDF-2 plasmid vector. The FBW7-Skp1 complex was purified in a similar manner to FIR Δexon2. In short, a competent cell transformed with the pCDF-2 Nus-tag-fused FBW7-Skp1 vector was cultured in LB medium. After resuspension of the cell pellet, the bacterial membrane was disrupted by ultrasonic homogenizer. The protein purification was performed by the Ni-affinity column, followed by Nus-tag cleavage with HRV-3C, Co affinity column, and gel filtration.

### Immunohistochemical staining

Thin slices were made from a paraffin block of esophageal cancer tissue and mounted on a glass slide. After deparaffinization, sections were incubated with antigen activator (Dako S2031, Tokyo, JAPAN) with 10-fold dilution for 40 min at 100°C. Sections then were left at room temperature for 20 min. After washing with water, sections were kept in distilled water for 5 min. The sections, topped with 3% hydrogen peroxide containing methanol (Dako S2023, Tokyo, JAPAN), were kept at room temperature for 30 min followed by washing with wash buffer (Dako S3006, Tokyo, JAPAN) three times at 5 min per washing, followed by incubation with primary antibody diluted with dilution buffer (Dako S0809, Tokyo, JAPAN) for 60 min at 37°C or overnight at 4°C. After incubation with primary antibody, sections were washed three times at 5 min per washing with the Wash Buffer (Dako S0809, Tokyo, JAPAN). Secondary antibody (ENVISION+/HRP-labeled Dako K5007, Tokyo, JAPAN) was added and sections were incubated for 60 min at 37°C. After incubation with diaminobenzidine (DAB; Dako K3468, Tokyo, JAPAN) for color development, sections were washed and kept in distilled water for 5 min. After nuclear staining with hematoxylin for 1 min, sections were washed with running water followed by dehydration, cleared, and sealed with a cover glass. The sections were examined under an optical microscope.

### CDDP and 5-fluorouracil (5FU) treatment

The DNA-damaging agent, CDDP, and 5FU sulfate powder from *Streptomyces verticillus* were purchased from Sigma-Aldrich (Tokyo, Japan; CDDP Lot no. MKBR4627V; PCode1001806142, 5FU Lot no. 083K0992), dissolved in distilled H_2_O at concentrations of 1 and 10 mg/mL, and stored at −20°C. HeLa, TTn, TE1, TE2, YES2, and YES3 cells were seeded in 6-well plates at a density of 1 to 4 × 10^6^ cells/well in 2 mL of culture medium. The cells were incubated at 37°C/5% CO_2_ until confluence (approximately 24 h). Immediately before drug treatment, the incubation medium was removed and replaced with fresh culture medium. Cells were treated with each cell’s half maximal inhibitory concentration (IC50) and to weaken the consistency of IC50. IC50 was determined by MTS assays.

### Small interfering RNAs (siRNAs) against FIR, SAP155, truncated FBW7 RNA plasmid

FIR and SAP155 siRNA duplexes and FBW7 plasmid were purchased from Sigma-Aldrich (Supplementary Table 4). The target sequences for FIR siRNA and SAP155 siRNA oligonucleotides are listed (Supplementary Table 4). Transient transfection of siRNA was done using Lipofectamine 2000 (Invitrogen) according to the manufacturer’s instructions. The transfected cells were cultured for 72 h at 37°C in a CO_2_ incubator.

### Display of three-dimensional structure of FBW7

To examine the possibility of molecular interaction between FBW7 and FIRs from structural viewpoint, two crystal structures, 2QVR and 2PEH, were downloaded from protein data bank (PDB).

### Statistical analysis

All statistical analyses were done using Prism6 produced by Graph Pad Software or StatFlex software version 6.0 (Artech, Osaka, Japan). *P* values were calculated by Student’s *t*-test. A value of *P* < 0.05 was considered statistically significant.

### AlphaLISA

AlphaLISA analysis for detecting anti-FIRs autoantibodies (IgG) was performed as described previously [23]. Briefly, AlphaLISA was performed in 384-well microtiter plates (white opaque OptiPlate^™^, Perkin Elmer, Waltham, MA, USA) containing 2.5 μL of 1:100-diluted serum and 2.5 μL of GST-fusion antigen proteins (10 μg/mL) in AlphaLISA buffer (25 mM HEPES, pH 7.4, 0.1% casein, 0.5% Triton X-100, 1 mg/mL Dextran 500, and 0.05% Proclin 300). The reaction mixture was incubated at room temperature for 6–8 h, mixed with anti-human IgG-conjugated acceptor beads (2.5 μL at 40 μg/mL), and glutathione-conjugated donor beads (2.5 μL at 40 μg/mL), and then incubated for seven days at room temperature in the dark. The chemical emission was read on an EnSpire Alpha microplate reader (PerkinElmer). Specific reactions were calculated by subtracting Alpha values of GST control from the values of GST-fusion proteins. The list of esophageal cancer patients and the results of their anti-FIRΔexon2s autoantibodies with other tumor markers are indicated (Supplementary Table 4). The cut off value of anti-FIRΔexon2 autoantibodies (IgG) was 3,171 counts indicated by +2 S.D. of mean of healthy group (Table 1 and Supplementary Table 5).

### Isothermal titration calorimetry (ITC) measurement

Isothermal titration calorimetry (ITC) measurement was performed to examine the interaction between FIR Δexon2 and FBW7. The experiment using MicroCal VP-ITC system was carried out at 30°C. The sample cell was filled with 50 mM phosphate buffer, pH 7.4, containing 15 μM purified Skp1-FWB7 complex. The solution containing 321 μM FIR Δexon2 was injected into sample cell from the syringe for titration. The injection volumes were 10 μL each, injection time was 20s, and a 150s delay was set between each injection.

## SUPPLEMENTARY MATERIALS FIGURES AND TABLES










