# Supplementary material for: Disturbed alternative splicing of FIR (PUF60) directed cyclin E overexpression in esophageal cancers
Source: Oncotarget. 2018 May 1;9(33):22929–44. doi: 10.18632/oncotarget.25149 (PMC5955432; doi:10.18632/oncotarget.25149)
Supplement: Supplementary file 1 [file oncotarget-09-22929-s001.pdf]

Disturbed alternative splicing of FIR (PUF60) directed cyclin E overexpression in esophageal cancers

SUPPLEMENTARY MATERIALS

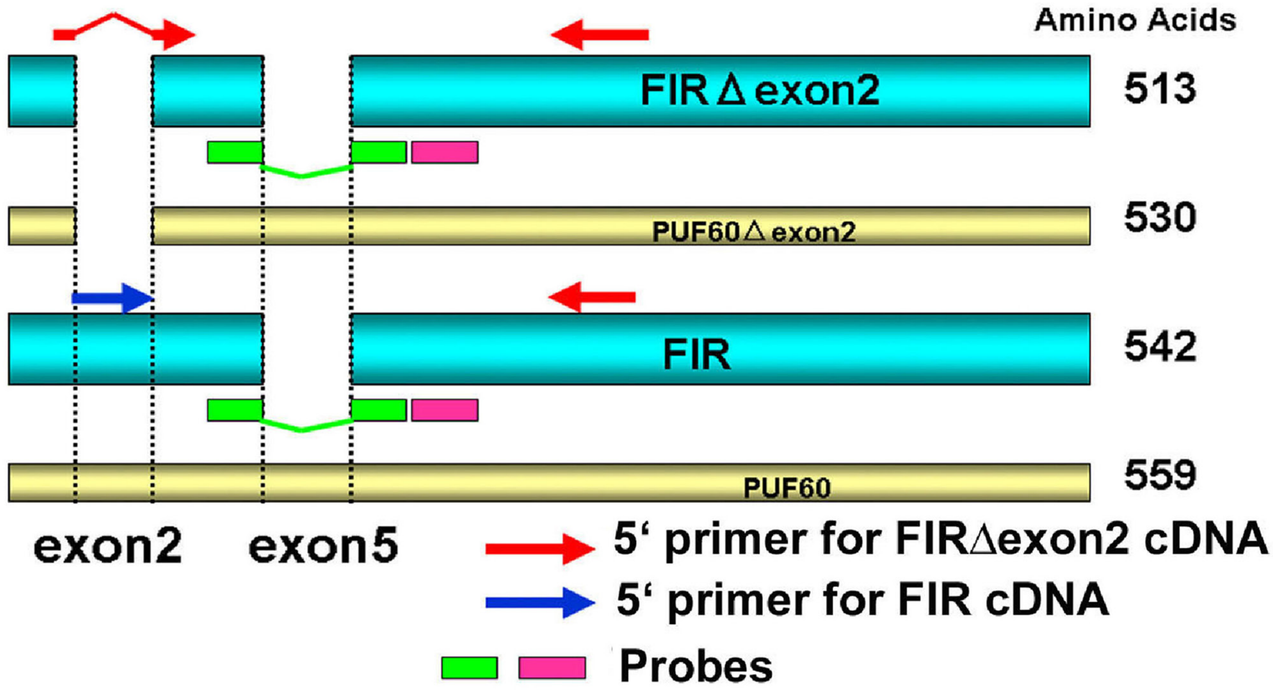

Supplementary Figure 1: Specific primers and probes for detecting FIR/FIRΔexon2 mRNA by qRT-PCR.

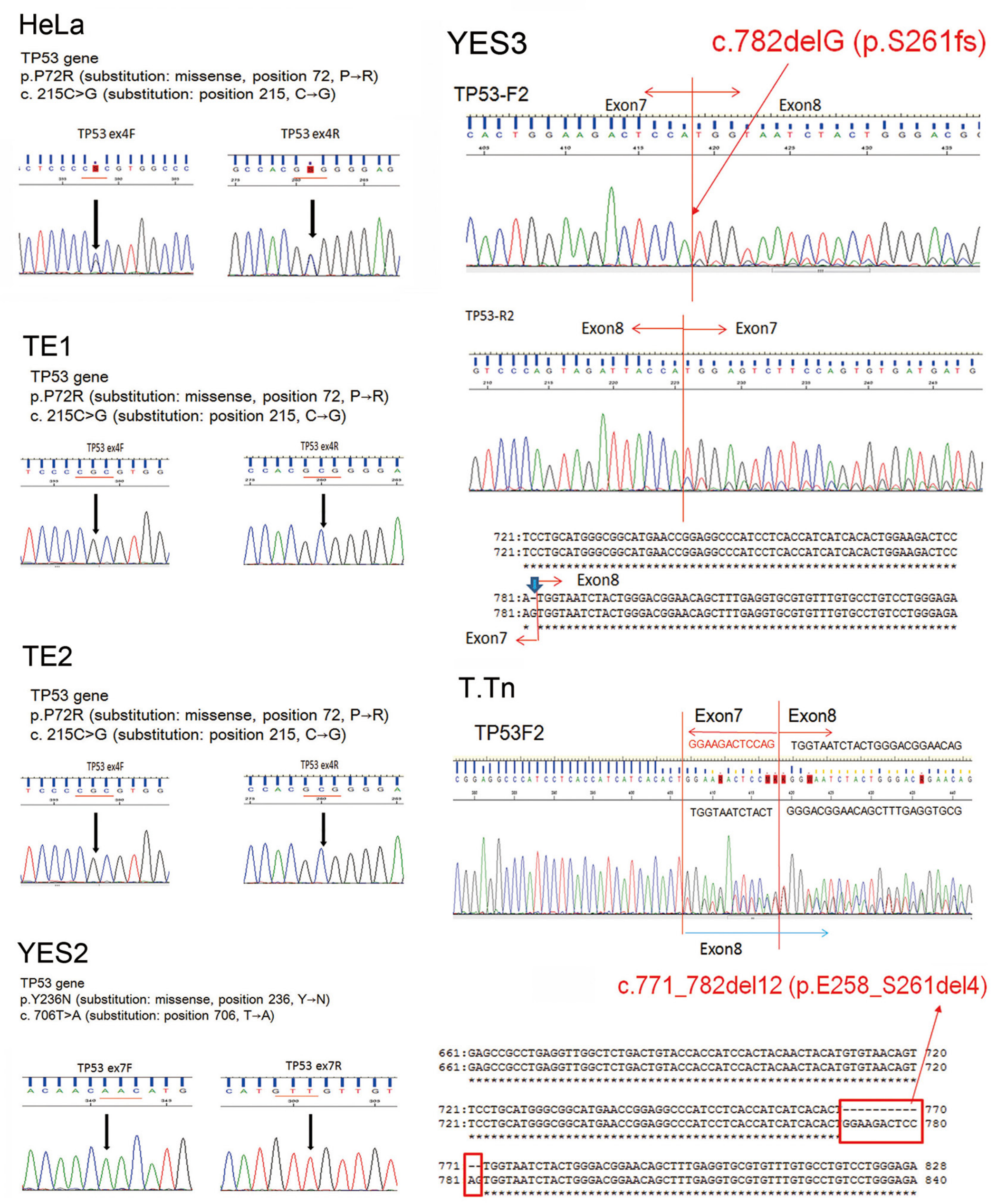

	Homo sapiens tumor protein p53		COSMIC
HeLa	NM_000546.5:c.215C>G	p.P72R	benign
TE1	c.215C>G	p.P72R	benign
TE2	c.215C>G	p.P72R	benign
YES2	c.706T>A	p.Y236N	pathogenic
YES3	c.782delG,c.782_783ins343	p.S261fs	pathogenic
T,Tn	c.771_782del12	p.E258_S261del4	pathogenic

Supplementary Figure 2: TP53 gene variations detected in ESCC cells examined in this study. TP53 genes of HeLa (human cervical squamous cell carcinoma), TE1 and TE2 were benign variation whereas those of YES2, YES3 and T.Tn were pathogenic variations. TE1, TE2, YES2, YES3 and T.Tn were esophageal squamous cell carcinoma cells.

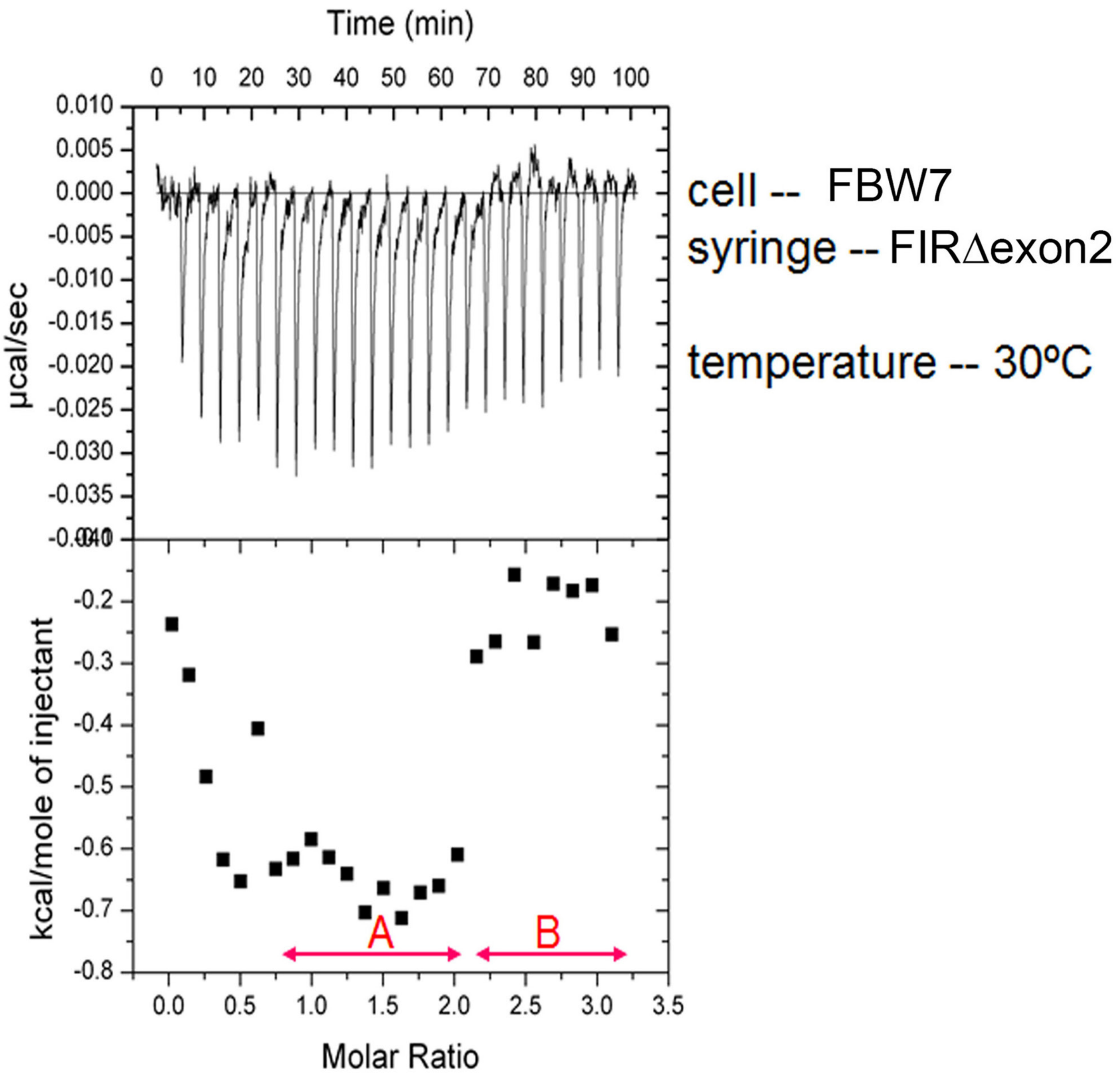

Supplementary Figure 3: ***In vitro*** **binding affinity between FIRΔexon2 and FBW7.** The titration curve of FBW7 with FIRΔexon2 suggested the molecular interaction between two proteins. The exothermic peaks were observed in the initial 17 injections. The peak level was decreased in the later injections (range A). Due to the sudden upward change in ITC thermogram (range B), it will be over 10^8 M^{-1}. Since the binding reaction is exothermic, the binding of FBW7 and FIRΔexon2 is enthalpically driven. This result is compatible with the importance of Asp of FBW7 in the molecular binding, because Asp usually contributes to hydrophilic interaction such as hydrogen bond formation between protein molecules.

Supplementary Table 1: Definition of stages of ESCC patients in this study according to Japanese classification of esophageal cancer.

See Supplementary File 1

Supplementary Table 2: Antibody lists used in this study.

See Supplementary File 2

Supplementary Table 3: The PCR primers and qRT-PCR conditions used in this study.

See Supplementary File 3

Supplementary Table 4 : FIR (PUF60) total, FIR, FIRΔexon2 and SAP155 siRNA sequence used in this study

siRNA (targets)	Probe	Sequence
FIR total siRNA	PUF60-1_s	5'rCrArGUrGrAUrGrGUUrCUrGrCrGrCrArATT3'
	PUF60-1_as	5'UUrGrCrGrCrArGrArArCrCrAUrCrArCUrGTT3'
	PUF60-2_s	5'rCrCrCUrArAUrGrGrCrCrGrCUrGrGUUTT3'
	PUF60-2_as	5'rArArCrCrArGrCrGrGrCrCrAUUrGrArGrGrGtt3'
FIR siRNA	FIR_s	5'rGrArCrArCrAUrGrGUrGrAUrGrCrArGrArATT3'
	FIR_as	5'UUrCUrGrCrAUrCrArCrCrAUrGUrGUrCTT3'
FIRΔexon2 siRNA	FIRΔexon2_s	5'rGrArCrCrAUrArGrCUrCUrCrGrGrCrArCrATT3'
	FIRΔexon2_as	5'UrGUrGrCrCrGrArGrArGrCUrAUrGrGUrCTT3'
SAP155 siRNA	SAP155-1_s	5'rCrGrArGUUUrGrCUUrGrGUrCrArGrArATT3'
	SAP155-1_as	5'UUrCUrGrArCrCrArArGrCrArArArCUrCrGTT3'
	SAP155-2_s	5'rGrArCUrAUUrCrAUrCrAUrCUrArCrGrATT3'
	SAP155-2_as	5'UrCrGUrArGrAUrGrAUrGrArAUrArGUrCTT3'

Supplementary Table 5: List of ESCC patients examined for anti-FIRΔexon2 autoantibodies in the sera. The cut off value of anti-FIRΔexon2 autoantibodies was 3,171 counts indicated by AlphaLISA counts.

See Supplementary File 4
